# Rupture of Axillary Artery during Reduction of Luxation of the Humerus; Diffuse Traumatic Aneurism; Amputation at the Shoulder-Joint; Death; Remarks

**Published:** 1880-12

**Authors:** 


RUPTURE OF AXILLARY ARTERY DURING REDUCTION OF LUXATION OF THE HUMERUS; DIFFUSE TRAUMATIC ANEURISM; AMPUTATION AT THE SHOULDER-JOINT; DEATH; REMARKS.

THE following interesting case is published from Charing-cross Hospital, by Mr. Bellamy:

Rupture of the axillary artery associated with simple dislocation of the humerus, or occurring during the reduction, is an extremely serious accident, though, happily, rare. In one case in which dislocation of the humerus was complicated by diffuse traumatic axillary aneurism, Mr. R. Adams reduced the dislocation, and then ligatured the subclavian artery. The patient recovered. Most of the cases of rupture of axillary artery during the attempts at reduction have been in dislocation of some weeks' duration. Indeed, it is probable that the rupture of the artery is due to laceration of the adhesions formed around the displaced bone in the neighborhood of the vessels. In the subjoined case no attempt at reduction seems to have been made for at least six weeks. In that time firm adhesions had doubtless formed. If the statement of the patient may be trusted, the rupture of the axillary occurred during the reduction. Unfortunately the mode of reduction is not stated. When admitted into Charing-cross Hospital there was only one alternative; the man must be left to bleed to death or amputation must be performed through the shoulder-joint. For the following notes we are indebted to Mr. Whitehead, surgical registrar: J. H.—— aged fifty-five, dislocated his right humerus on Dec. 16th, 1879; and in the following month he went to a large metropolitan hospital, where the house-surgeon put him under an anæsthetic and attempted reduction. On Feb. 2d, one of the surgeons made further attempts, and the patient stated that he immediately became aware of a swelling in the armpit. A fortnight after this he was discharged. He had lost sensation since the time of the last attempt. Three weeks after he left the hospital a little bright blood oozed through the skin, and on July 30th he had more severe hemorrhage, losing about quarter of a pint of arterial blood, and then the bleeding ceased. On admission, situated in the region of the right shoulder was a large tumour. The walls were very tense, and pulsating. The clavicle was distinct, and not involved. The pulsation was best felt at the posterior part of the axilla. The arm below was much swollen and waxy; no pulse could be felt at the wrist. At the lower part of the tumour, in the axilla, the skin was dark and sloughy, from which flowed a watery discharge. The tumour was not distinctly circumscribed at its lower margin, and was about as large as a man's head. The patient was anxious in appearance, and stated that he had been rapidly losing health since the appearance of the tumour. He had had no attacks of syncope or breathlessness, and the face was not markedly blanched. Pulse 96; respiration 18. Conjunctiva and mucous membrane pale and anæmic. He had slight cough and watery expectoration. After consultation with Mr. Amphlett (who admitted the case from his out-patients) and his other colleagues, Mr. Bellamy determined on amputation through the shoulder-joint. This was done by commencing with an anterior flap, from without and *not* transfixing. Although the subclavian artery was most efficiently compressed by Mr. Bloxam, the hemorrhage on the first sweep of the knife was terrific, the blood coming from the tense sac and from the enlarged vessels behind and below. The second or posterior flap was cut after disarticulation, in a few seconds, and some pounds of clot and organized lymph were removed. The main trunk, as far as its topographical position should have been, was not recognizable. There was a hard cartilaginous-like tube on the wall of the thorax, throwing a fearful jet of blood, and which, notwithstanding the pressure on the subclavian artery, was secured after great difficulty. After all bleeding points had been secured the flaps were adjusted and the patient removed to bed. Unfortunately, he never rallied, and about an hour afterwards died.—*The Lancet.*

